# A fiber-enriched diet alleviates *Staphylococcus aureus*-induced mastitis by activating the HDAC3-mediated antimicrobial program in macrophages via butyrate production in mice

**DOI:** 10.1371/journal.ppat.1011108

**Published:** 2023-01-19

**Authors:** Caijun Zhao, Lijuan Bao, Yihong Zhao, Keyi Wu, Min Qiu, Lianjun Feng, Naisheng Zhang, Xiaoyu Hu, Yunhe Fu

**Affiliations:** Department of Clinical Veterinary Medicine, College of Veterinary Medicine, Jilin University, Changchun, Jilin Province, China; University of Michigan, UNITED STATES

## Abstract

Mounting evidence suggests that the gut microbiota plays an important role in the pathogenesis of mastitis, an important disease affecting the health of lactating women and the development of the dairy industry. However, the effect of the regulation of the gut microbiota by dietary components on mastitis development remains unknown. In this study, we found that a fiber-enriched diet alleviated *Staphylococcus aureus* (*S*. *au*)-induced mastitis in mice, which was dependent on the gut microbiota as depletion of the gut microbiota by antibiotics abolished this protective effect. Likewise, fecal microbiota transplantation (FMT) from high-inulin (HI)-treated mice (HIF) to recipient mice improved *S*. *au-*induced mastitis in mice. Consumption of an HI diet and HIF increased fecal short-chain fatty acid (SCFA) levels compared with the control group. Moreover, treatment with SCFAs, especially butyrate, alleviated *S*. *au-*induced mastitis in mice. Mechanistically, consumption of an HI diet enhanced the host antimicrobial program in macrophages through inhibiting histone deacetylase 3 by the production of butyrate. Collectively, our results suggest that modulation of the gut microbiota and its metabolism by dietary components is a potential strategy for mastitis intervention and serve as a basis for other infectious diseases.

## Author summary

*S*. *au* infection is a common and serious disease threatening humans and animals. Emerging evidence has supported the protective effects of the gut microbiota in regulating host susceptibility to pathogens. However, whether and how modulation of the gut microbiota by dietary components contributes to protection against pathogen invasion in distal organs remain unknown. Here, using an *S*. *au*-induced mouse mastitis model, we found that consumption of an HI-enriched diet alleviated *S*. *au*-induced mastitis in mice, and that this effect was dependent on the gut microbiota as the depletion of commensal microbiota reversed the protective effect of an HI-enriched diet. The role of the gut microbiota in HI diet-mediated effects was also confirmed by FMT. We further observed that treatment with an HI diet increased fecal SCFA levels, which can alleviate *S*. *au*-induced mastitis in mice by activating host antimicrobial programs in macrophages through inhibition of histone deacetylase 3. Our research indicates the important role of gut microbiota and its metabolism in protecting against *S*. *au* infection in distal organs and serves as a basis for other infectious diseases.

## Introduction

Mastitis is one of the most common diseases for lactating women and female animals, which increases the risk for breast cancer for women and impairs production performance for dairy cows [1,2]. Although pathogen invasion, especially *Staphylococcus aureus* (*S*. *au*) and *Escherichia coli* (*E*. *coli*), has been commonly thought to be the major cause of mastitis [3,4], emerging evidence also indicates that the gut microbiota plays an important role in the pathogenesis of mastitis [5–8]. Our previous studies revealed that depletion of the commensal gut microbiota contribute to initial mastitis and facilitate the development of mastitis caused by *S*. *au* and *E*. *coli* [5,6]. In contrast, aggravated mastitis symptoms caused by gut dysbiosis were improved after fecal microbiota transplantation (FMT) from control mice [5]. Ruminal dysbiosis caused by high-concentrate diet-induced subacute ruminal acidosis can also increase somatic cell count (SCC) in milk and trigger mastitis in dairy cows [7]. Likewise, Ma et al., found that FMT from mastitis cows, but not healthy cows, to mice induced mastitis symptom in mice that were then alleviated by supplementation with *Lactobacillus* [8]. Interestingly, our previous study also demonstrated that consumption of a tryptophan-enriched diet can limit *E*. *coli*-induced mastitis in mice by activating the aryl hydrocarbon receptor via gut microbiota metabolism [6]. These findings confirm the essential role of the gut microbiota in regulating the development and outcome of mastitis; however, whether regulation of the gut microbiota can alleviate pathogen-induced mastitis remains unknown.

As one of the most important substrates for short-chain fatty acid (SCFA) production, dietary fiber has multiple beneficial effects on host homeostasis and disease development [9,10]. For example, a fiber-enriched diet modulates skin barrier integrity by promoting keratinocyte metabolism and differentiation through production of SCFAs via the gut microbiota [11]. Increased dietary fiber consumption alleviates type 2 diabetes by selectively promoting gut microbes in humans [12]. Higher intake of whole grains and dietary fiber has also been associated with a lower risk of liver cancer and chronic liver disease mortality [13] and improved intestinal inflammation [14]. In contrast, deficiency of dietary fiber and insufficient activation of SCFAs sensing receptors leads to cardiovascular disease [15]. In addition, deprivation of dietary fiber increases susceptibility to intestinal pathogens [16]. Notably, our previous study also showed that reduction of SCFA caused by the depletion of the gut microbiota also aggravates *S*. *au*-induced mastitis [5], but the underlying mechanism remains unclear. In addition to a direct influence on disease outcome, insufficient dietary fiber intake also impairs the recovery of the gut microbiota and its metabolism after antibiotic treatment [17]. These findings suggest the potential protective role of dietary fiber and its gut microbiota-derived metabolites in metabolic and inflammatory diseases; however, whether dietary fiber contributes to the prevention and treatment of *S*. *au*-induced mastitis and the underlying mechanisms are still unclear.

Given the important role of the gut microbiota and its metabolites in the development of mastitis [5–7] and the beneficial effects of dietary fiber in the regulation of the gut microbiota and disease outcome [12,16,18], the aim of this study was to investigate the protective effects of a fiber-enriched diet in distal organ infection using a *S*. *au*-induced mastitis model in mice. Our results showed that inulin, a classical soluble fiber [11], treatment alleviated *S*. *au*-induced mastitis in mice and this effect was dependent on the gut microbiota, as confirmed by commensal microbiota depletion and FMT. Consumption of an inulin-enriched diet significantly changed the gut microbiota and increased fecal SCFA levels, which enhanced the antimicrobial program in macrophages by inhibiting histone deacetylase (HDAC) 3. Taken together, our findings indicate that a fiber-enriched diet ameliorates *S*. *au*-induced mastitis in mice by modulating the gut microbiota, which suggests that regulation of the gut microbiota by dietary components is a potential strategy for the intervention of pathogen infection in distant organs.

## Results

### A high-inulin (HI) diet attenuates *S*. *au*-induced mastitis in mice

**Fig 1:**
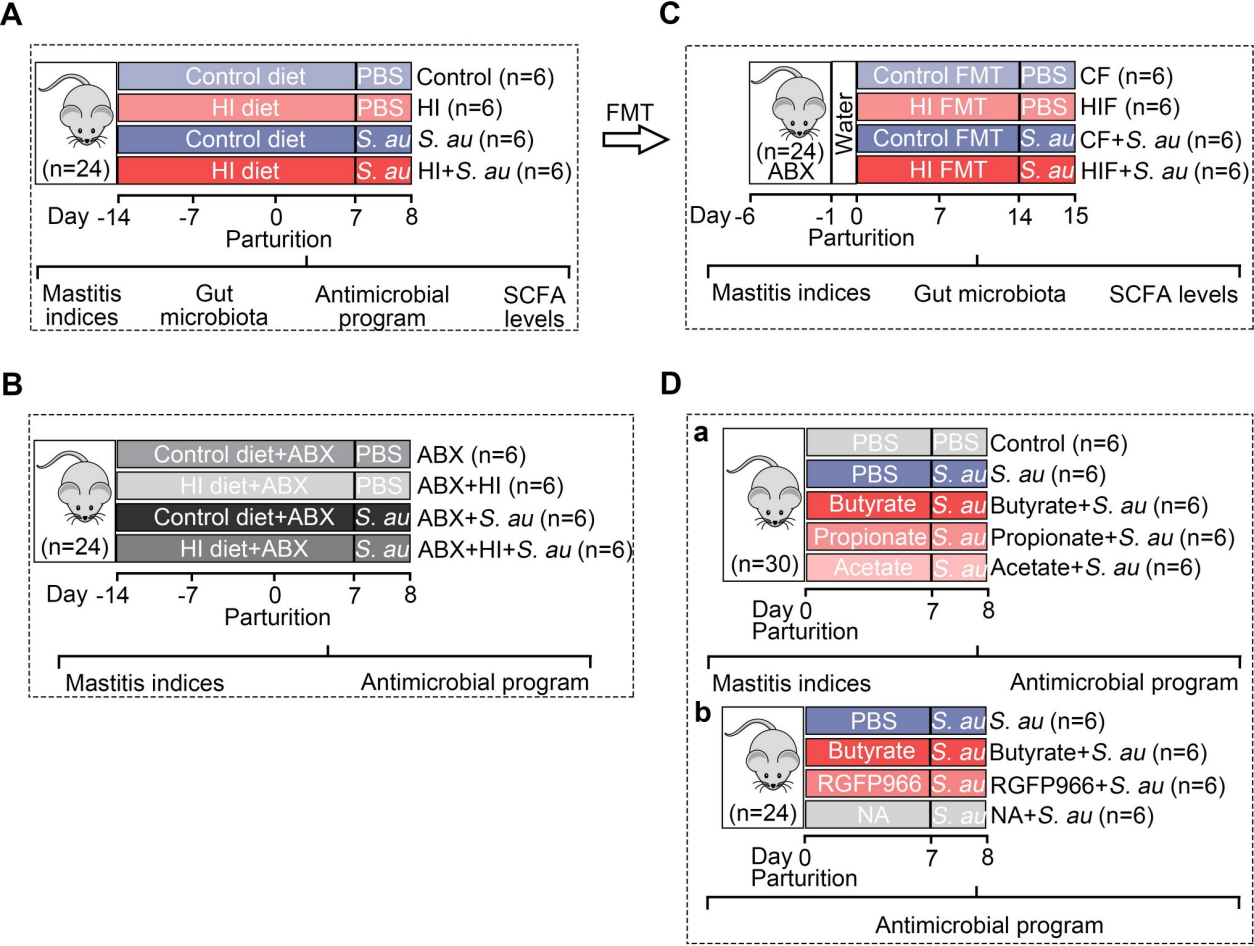
Experimental overview. **A.** The effects of a high-fiber diet on *S*. *au*-induced mastitis in mice were investigated. Mice were separated into four groups, including control, high-inulin (HI), *S*. *au* and HI + *S*. *au* groups (n = 6 per group), and fed with control or HI diet for three weeks followed by *S*. *au*-induced mastitis. **B**. The depletion of the gut microbiota by antibiotic cocktail (ABX) was performed to investigate the role of gut microbiota in HI diet-mediated protective effects on *S*. *au*-induced mastitis (n = 6 per group). **C**. Fecal microbiota transplantation (FMT) from control- (CF) or HI diet- (HIF) treated mice was used to confirm the role of gut microbiota (n = 6 per group). **D**. The effects of butyrate, propionate and acetate on *S*. *au*-induced mastitis were investigated (**a**) and HDAC inhibitors, including RGFP966 and NA, were used to investigate the underlying mechanism (**b**) (n = 6 per group).

**Fig 2:**
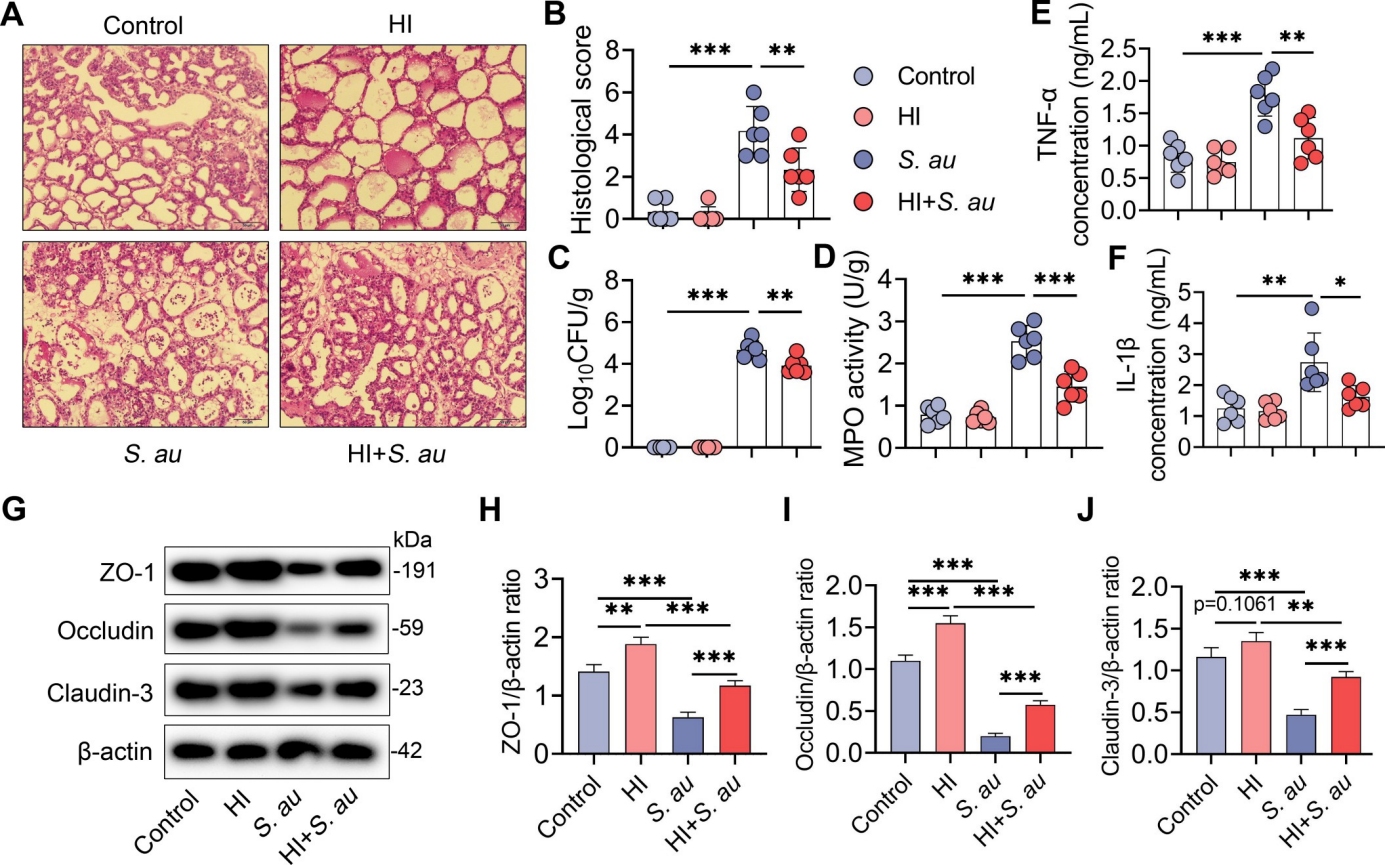
A high-inulin diet attenuates *S*. *au*-induced mastitis in mice. Mice that were pregnant for a week were randomly divided into four groups, and fed an AIN93G diet (control) or high-inulin diet (AIN93G + 20% inulin, HI) for three weeks followed by the *S*. *au*-induced mastitis model (*S*. *au* and HI + *S*. *au* groups) (n = 6 per group). **A**. Representative H&E-stained images of the mammary glands from different treatment groups (scale bar, 50 μm). **B**. Histological score of the mammary gland based on H&E-stained sections (n = 6). **C**. Mammary bacterial burden from the indicated mice (n = 6). **D-F**. Inflammatory markers, including MPO activity (**D**), TNF-α (**E**) and IL-β (**F**), from the indicated groups (n = 6). **G**. Representative images of mammary tight junction (TJ) proteins, including ZO-1, Occludin and Claudin-3, by western blotting. **H-I**. Relative intensity of TJ proteins including ZO-1 (**H**), Occludin (**I**) and Claudin-3 (**J**) using β-actin as an endogenous control. Data are presented as the means ± SD (**B-F**, **H-J**) and one-way analysis of variance (ANOVA) was performed for statistical analysis (**B-F**, **H-J**). *p < 0.05, **p < 0.01 and *** *p* < 0.001 indicate significant differences.

The whole experimental design for this study was shown in **Fig 1**. We first investigated whether a high-fiber diet alleviates mastitis caused by *S*. *au* in mice (**Fig 1A**). The essential role of gut microbiota in high-fiber diet-mediated protective effect on *S*. *au*-induced mastitis was confirmed by antibiotic (ABX) treatment and FMT (**Fig 1B and 1C**). Based on the increased production of SCFA levels evaluated by a high-fiber diet, we then investigated the role of SCFA on *S*. *au*-induced mastitis in mice and focused on the antimicrobial program in macrophages (**Fig 1D**). To investigate the protective effects of an HI diet in pathogen-induced mastitis, mice were treated with 20% inulin for three weeks followed by *S*. *au* treatment. Histological analysis showed that *S*. *au* treatment induced significant inflammatory cell infiltration and barrier injury compared with the control group (**Fig 2A** and **2B**), while HI diet supplementation reduced the increase in leucocytes in mammary acini caused by *S*. *au* (**Fig 2A** and **2B**). In addition, an HI diet treatment reduced mammary bacterial burden compared with a control diet upon *S*. *au* infection in the mammary gland (**Fig 2C**). Consistently, mice in the HI diet group had lower levels of proinflammatory markers, including MPO activity (**Fig 2D**), tumor necrosis factor (TNF)-α (**Fig 2E**) and interleukin (IL)-1β (**Fig 2F**) in the mammary gland than the *S*. *au* group. Similar to our previous finding that pathogens can damage the blood-milk barrier [5,6], we showed that *S*. *au* treatment reduced the mammary tight junction (TJ) proteins ZO-1, Occludin and Claudin-3 compared with those of the control group, and that these decreases were improved by HI diet supplementation (**Fig 2G–2J**). These results indicate that an HI diet alleviates *S*. *au*-induced mastitis in mice.

### The alleviation of *S*. *au*-induced mastitis by an HI diet is dependent on the gut microbiota

**Fig 3:**
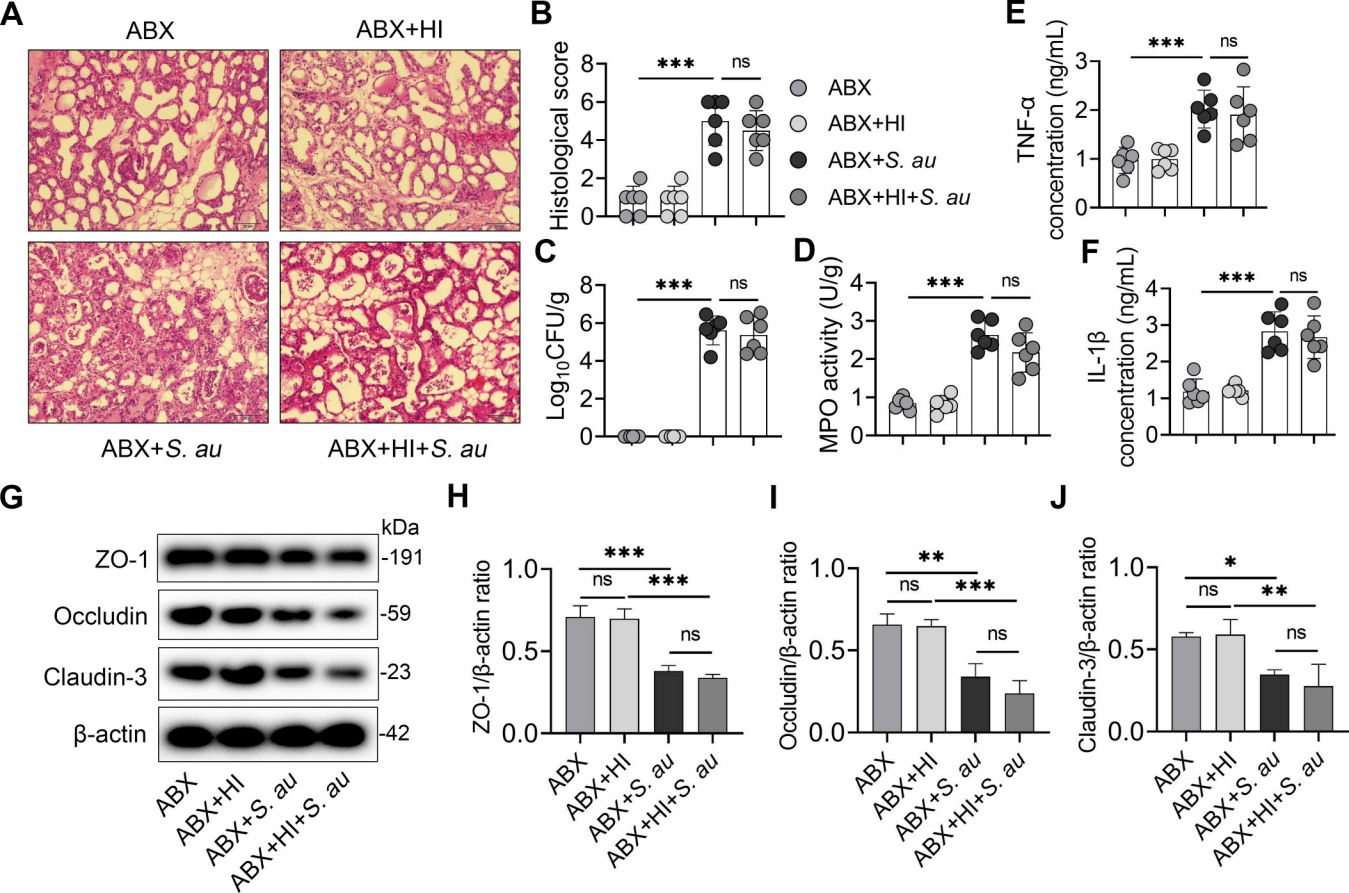
Depletion of the gut microbiota impairs HI diet-mediated protective effects on *S*. *au*-induced mastitis in mice. Mice were treated with a control diet supplemented with or without 20% inulin for three weeks under the condition of gut microbiota depletion by a cocktail of antibiotics (ABX) followed by *S*. *au*-induced mastitis (n = 6 per group). **A**. Representative H&E-stained images of the mammary glands from different treatment groups (scale bar, 50 μm). **B**. Histological score of the mammary gland based on H&E-stained sections (n = 6). **C**. Mammary bacterial burden from the indicated mice (n = 6). **D-F**. Inflammatory markers including MPO activity (**D**), TNF-α (**E**) and IL-β (**F**) from the indicated groups (n = 6). **G**. Representative images of mammary TJ proteins, including ZO-1, Occludin and Claudin-3, by western blotting. **H-I**. Relative intensity of TJ proteins including ZO-1 (**H**), Occludin (**I**) and Claudin-3 (**J**), using β-actin as an endogenous control. Data are presented as the means ± SD (**B-F**, **H-J**) and one-way ANOVA was performed for statistical analysis (**B-F**, **H-J**). *p < 0.05, **p < 0.01 and *** *p* < 0.001 indicate significant differences.

Mounting evidence has indicated that the gut microbiota play an essential role in the digestion and metabolism of dietary fiber [19,20]. We therefore investigated whether the protective role of an HI diet in *S*. *au*-induced mastitis was dependent on the gut microbiota by depletion of the commensal microbiota via treatment with an antibiotic cocktail. Consistent with our previous findings [5], gut-dysbiotic mice displayed significant mammary injury and damage upon *S*. *au* infection (**Fig 3A and 3B**). However, different from mice in the HI + *S*. *au* group, mice in the ABX + HI + *S*. *au* group showed similar mammary injury and inflammation compared with those of the ABX+ *S*. *au* group (**Fig 3A and 3B**). Likewise, depletion of the commensal microbiota by ABX eliminated the protective effect of an HI diet on the mammary *S*. *au* burden (**Fig 3C**). Furthermore, *S*. *au*-treated gut-dysbiotic mice had increased mammary inflammatory markers, including MPO activity (**Fig 3D**), TNF-α (**Fig 3E**) and IL-1β (**Fig 3F**), compared with the ABX group. However, no significant differences were detected between the ABX + HI + *S*. *au* group and the ABX+ *S*. *au* group (**Fig 3D–3F**), which suggests that ABX abolished the protective effect of an HI diet on the decreases in inflammatory markers caused by *S*. *au* in mice. Additionally, mice in the ABX + *S*. *au* group had lower levels of the TJ proteins ZO-1, Occludin and Claudin-3 than those in the ABX group (**Fig 3G–3J**), while no significant changes were observed after HI diet supplementation in gut-dysbiotic mice (**Fig 3G–3J**). Collectively, these results indicate that the protective effects of an HI diet on *S*. *au*-induced mastitis are dependent on the gut microbiota in mice.

### An HI diet alters the gut microbiota in mice

**Fig 4:**
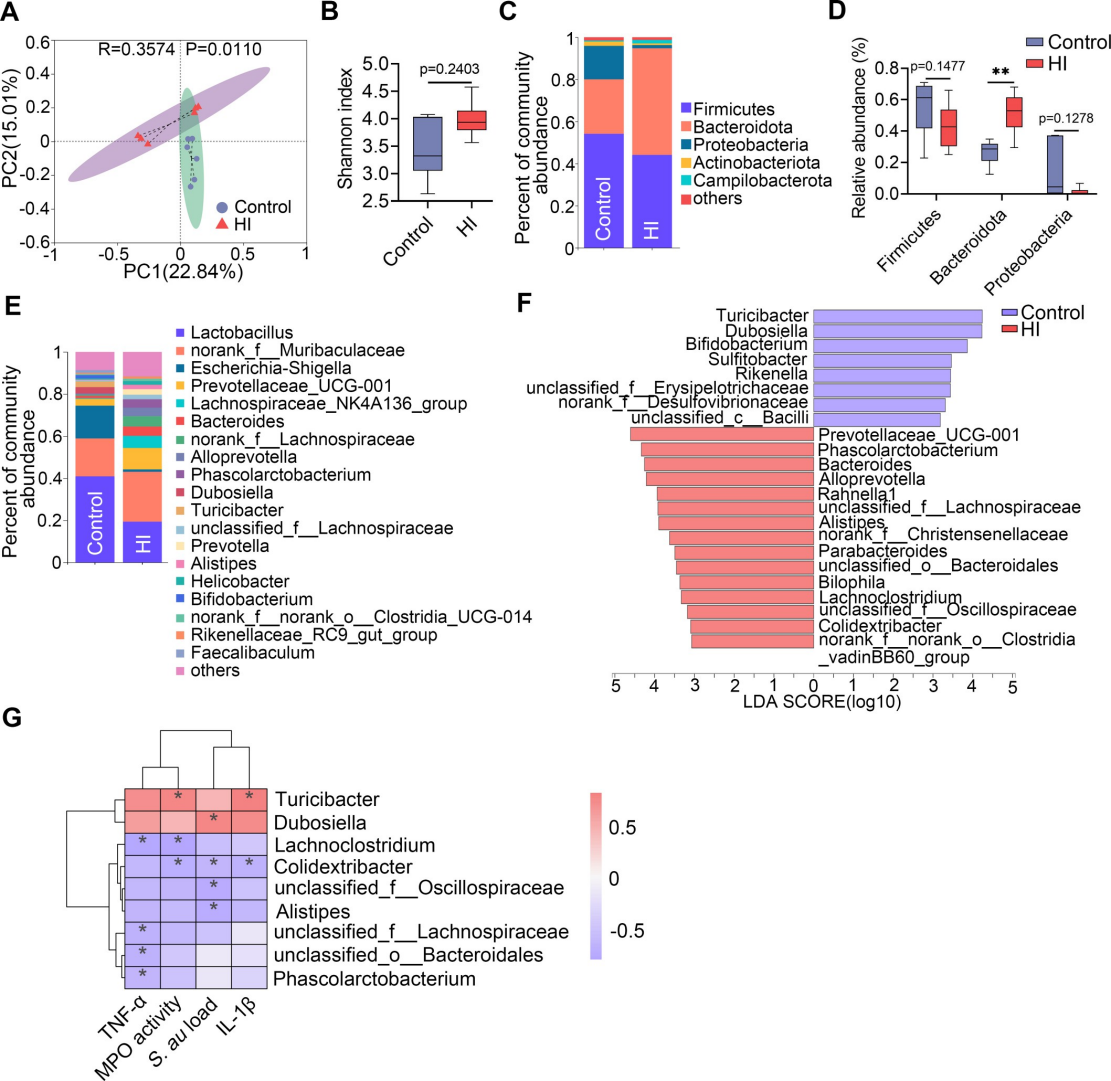
HI diet alters the gut microbiota in mice. Mice were fed a control diet or HI diet for three weeks and the gut microbiota were analyzed by 16S rRNA sequencing. **A.** PCoA showed different gut microbial structure between the control and HI groups based on unweighted UniFrac distance (R = 0.3574, P = 0.0110, n = 6). **B**. Shannon index from the indicated groups (n = 6). **C**. Top five gut microbial compositions at the phylum level from different treatment groups. **D**. Relative abundances of selected bacterial taxa in the gut microbiota from different groups (n = 6). **E**. Bacterial composition at the genus level in the indicated groups. **F**. LEfSe showed different bacterial taxa that were enriched in different groups (log10 LDA score > 3). **G**. Spearman correlation analysis between selected gut microbial taxa and mammary inflammatory parameters. Red indicates a positive correlation, and green indicates negative correlation. Data are expressed as boxplots and the Mann-Whitney *U* test was performed for statistical analysis (**B** and **D**). **p < 0.01 indicates a significant difference.

Next we investigated the effect of an HI diet on the gut microbiota in mice. Principal coordinate analysis (PCoA) score plots for the fecal samples showed that mice in the HI group had distinct microbial structures from those of the control group (R = 0.3574, P = 0.110) (**Fig 4A**). Alpha diversity analysis found that mice in the HI group had lightly increased alpha diversity compared with those in the control group (**Figs 4B** and S1A and S1B). At the phylum level, mice in the HI group had enhanced *Bacteroidota* and reduced *Proteobacteria* compared with the control group (**Fig 4C and 4D**). Consistently, different genera were identified between the HI and control groups at the genus level (**Fig 4E**). To identify the different taxa enriched in each group, linear discriminant analysis (LDA) effect size (LEfSe) was performed (LDA score (log10) > 3). A total of 15 genera were enriched in mice in the HI group, including *Prevotellaceae*_UCG-001, *Phascolarctobacterium*, *Bacteroides*, *Alloprevotella*, *unclassified_f__Lachnospiraceae*, *Alistipes*, *Parabacteroides*, *unclassified_o__Bacteroidales* and *unclassified_f__Oscillospiraceae*, compared with those of the control group (**Fig 4F**), while 8 other genera, including *Turicibacter* and *Dubosiella*, were depleted in the HI group (**Fig 4F**). Furthermore, we found that genera enriched in the HI group displayed a negative correlation with mammary *S*. *au* load and inflammatory parameters, while *Turicibacter* and *Dubosiella*, which were depleted in the HI group, showed a positive correlation with these mammary parameters (**Fig 4G**). Taken together, these results indicate that an HI diet significantly changed the gut microbiota, which contributed to protection against *S*. *au*-induced mastitis.

### FMT from an HI diet treatment improves *S*. *au*-induced mastitis in mice

**Fig 5:**
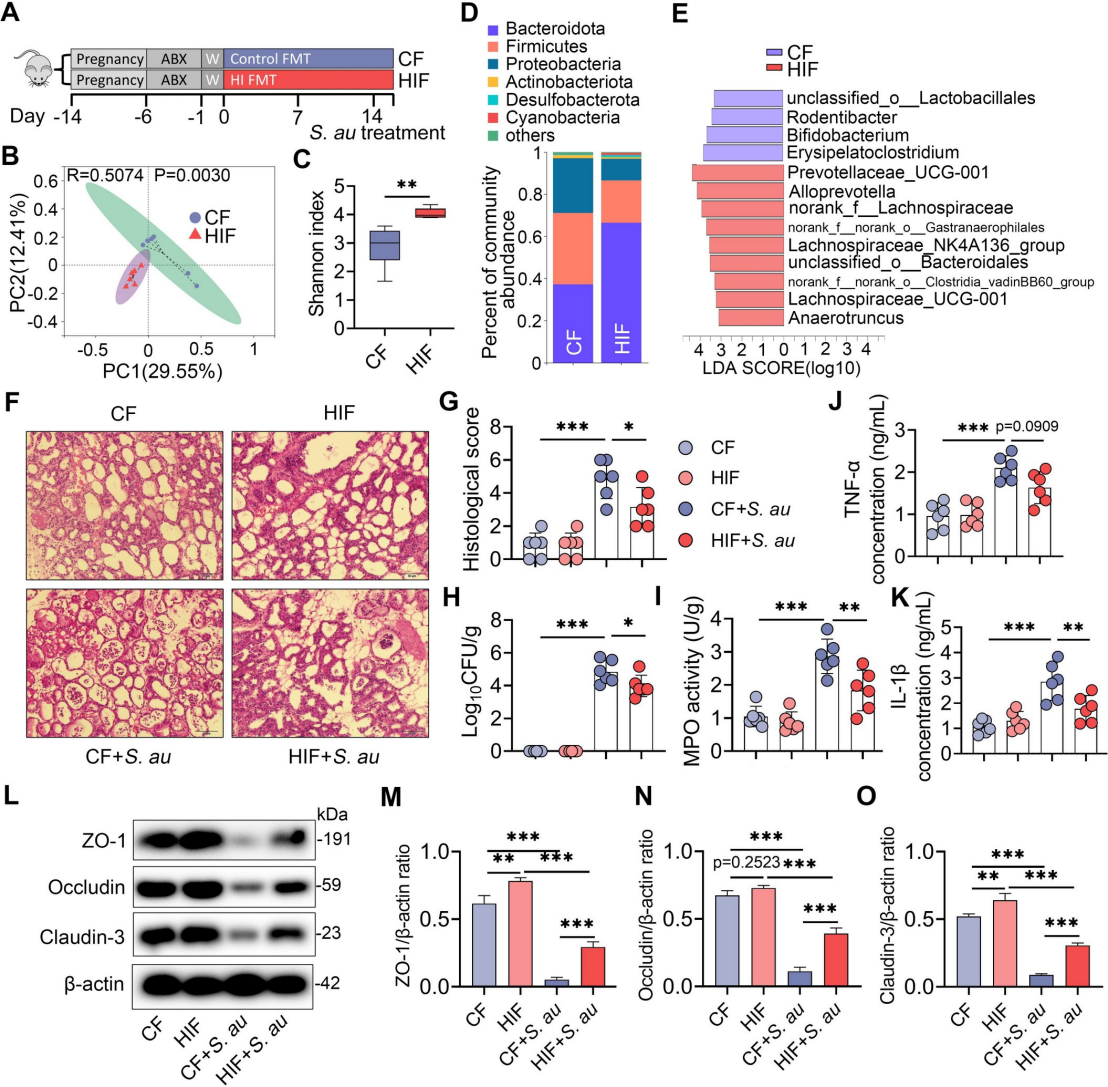
FMT from the HI diet treatment group improves *S*. *au*-induced mastitis in mice. **A.** Illustration of FMT. Pregnant mice were treated with ABX to deplete the commensal microbiota and then subjected to FMT from the control or HI treatment groups for two weeks (n = 6). **B**. PCoA showed different gut microbial structures between the CF and HIF groups based on unweighted UniFrac distance (R = 0.5074, P = 0.0030, n = 6). **C**. Shannon index from the indicated groups (n = 6). **D**. Gut microbial composition at the phylum level from different treatment groups (n = 6). **E**. LEfSe showed different bacterial taxa that were enriched in different groups (log10 LDA score > 3). **F**. Representative H&E-stained images of the mammary glands from different treatment groups (scale bar, 50 μm). **G**. Histological score of the mammary gland based on H&E-stained sections (n = 6). **H**. Mammary bacterial burden from the indicated groups (n = 6). **I-K**. Inflammatory markers, including MPO activity (**I**), TNF-α (**J**) and IL-β (**K**), from the indicated groups (n = 6). **L**. Representative images of mammary TJ proteins, including ZO-1, Occludin and Claudin-3, by western blotting. **M-O**. Relative expressions of TJ proteins, including ZO-1 (**M**), Occludin (**N**) and Claudin-3 (**O**) were shown using β-actin as an endogenous control. Data are expressed as boxplots (**C**) or means ± SD (**G-K**, **M-O**), and the Mann-Whitney *U* test (**C**) and one-way ANOVA (**G-K**, **M-O**) were performed for statistical analysis. *p < 0.05, **p < 0.01 and *** *p* < 0.001 indicate significant differences.

To confirm the role of the gut microbiota in the HI diet-mediated protective effects against *S*. *au*-induced mastitis in mice, FMT from the control (CF) and HI diet (HIF) treatment groups was performed (**Fig 5A**). PCoA showed that mice in the HIF group displayed different gut microbial structures from those in the CF group (**Fig 5B**). Consistent with the donor, mice in the HIF group had increased microbial diversity and richness compared with those in the CF group (**Figs 5C and** S2A and S2B). At the phylum and genus levels, mice in the HIF group showed different microbial compositions characterized by increased *Bacteroidota* and reduced *Proteobacteria* compared with those in the control group (**Figs 5D** and S2C). LEfSe also identified that *Prevotellaceae*_UCG-001, the most significantly enriched taxa in the HI group, was enriched in the HIF group (**Fig 5E**). These results indicate that FMT from the HI diet treatment group significantly reshaped the gut microbiota in recipient mice. Next, we found that mice in the HIF + *S*. *au* group displayed a lower degree of mammary inflammatory injury than those in the CF + *S*. *au* group (**Fig 5F and 5G**). Notably, a lower bacterial burden was also observed in the HIF + *S*. *au* group than in the CF + *S*. *au* group (**Fig 5H**). Consistently, HIF reduced mammary inflammatory markers, including MPO activity (**Fig 5I**) and TNF-α (**Fig 5J**) and IL-1β (**Fig 5K**) concentrations, compared with CF treatment following *S*. *au* infection. Similar to the HI diet treatment group, HIF also reversed the decreases in the mammary TJ proteins ZO-1, Occludin and Claudin-3 caused by *S*. *au* in mice (**Fig 5L–5O**). Collectively, these results suggest that the gut microbiota is responsible for the protective effects of an HI diet against *S*. *au*-induced mastitis in mice.

### An HI diet alleviates *S*. *au*-induced mastitis by increasing SCFA levels via the gut microbiota

**Fig 6:**
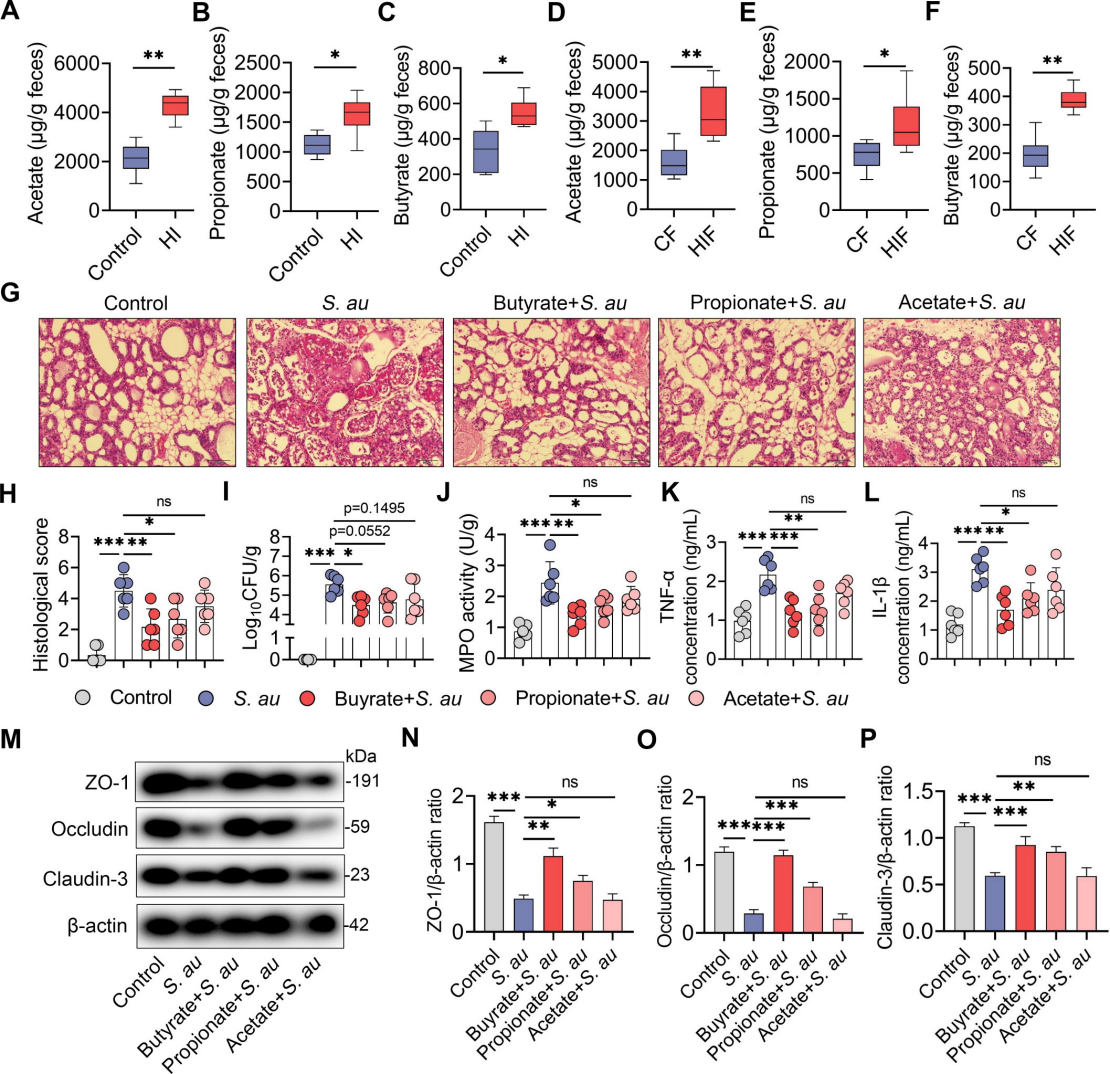
HI diet-derived SCFAs by gut microbiota metabolism alleviate *S*. *au*-induced mastitis in mice. **A-C**. Fecal acetate (**A**), propionate (**B**) and butyrate (**C**) levels from control diet- and HI diet-treated mice (n = 6). **D-F**. Fecal acetate (**D**), propionate (**E**) and butyrate (**F**) levels from different FMT groups (n = 6). **G-P**. Mice were treated with 30 mg/kg butyrate, propionate and acetate for a week, followed by *S*. *au*-induced mastitis (n = 6). **G**. Representative H&E-stained images of the mammary glands from different treatment groups (scale bar, 50 μm). **H**. Histological score of the mammary gland based on H&E-stained sections (n = 6). **I**. Mammary *S*. *au* burden from the indicated groups (n = 6). **J-L**. Inflammatory markers, including MPO activity (**J**), TNF-α (**K**) and IL-β (**L**), from the indicated groups (n = 6). **M**. Representative images of mammary TJ proteins, including ZO-1, Occludin and Claudin-3, by western blotting. **N-P**. Relative intensity of TJ proteins including ZO-1 (**N**), Occludin (**O)** and Claudin-3 (**P**), using β-actin as an endogenous control. Data are presented as boxplots (**A-F**) or means ± SD (**H-L** and **N-P**). The Mann-Whitney *U* test (**A-F**) and one-way ANOVA (**H-L** and **N-P**) were performed for statistical analysis. *p < 0.05, **p < 0.01 and *** *p* < 0.001 indicate significant differences.

Dietary fibers are responsible for the production of beneficial metabolites including short-chain fatty acids (SCFAs) by the gut microbiota [21,22]. We therefore investigated whether an HI diet protects against *S*. *au*-induced mastitis by producing SCFAs. As expected, mice in the HI group had increased fecal acetate, propionate and butyrate levels compared with the control group (**Fig 6A–6C**). Consistently, these increases were also observed in the HIF group compared with the CF group (**Fig 6D–6F**). We next studied the role of HI diet-derived SCFAs in the development of *S*. *au*-induced mastitis in mice. Consistent with our previous finding [5], we found that butyrate and propionate but not acetate significantly reduced *S*. *au*-induced mammary injury (**Fig 6G and 6H**). Notably, only butyrate treatment reduced the mammary *S*. *au* load compared with the *S*. *au* group (**Fig 6I**). Moreover, we found that butyrate and propionate treatments reduced mammary inflammatory MPO activity (**Fig 6J**) and TNF-α (**Fig 6K**) and IL-1β (**Fig 6L**) concentrations compared with the *S*. *au* group. However, no significant changes in these inflammatory markers were detected between the acetate treatment group and the *S*. *au* group (**Fig 6J–6L**). Consistently, mice in the butyrate and propionate treatment groups, but not the acetate treatment group, had increased mammary TJ proteins ZO-1, Occludin and Claudin-3 compared with those in the *S*. *au* group (**Fig 6M–6P**). These results indicate that HI diet-mediated SCFA production contributes to alleviating *S*. *au*-induced mastitis in mice.

### An HI diet activates macrophage antimicrobial defense through SCFA-mediated HDAC inhibition

**Fig 7:**
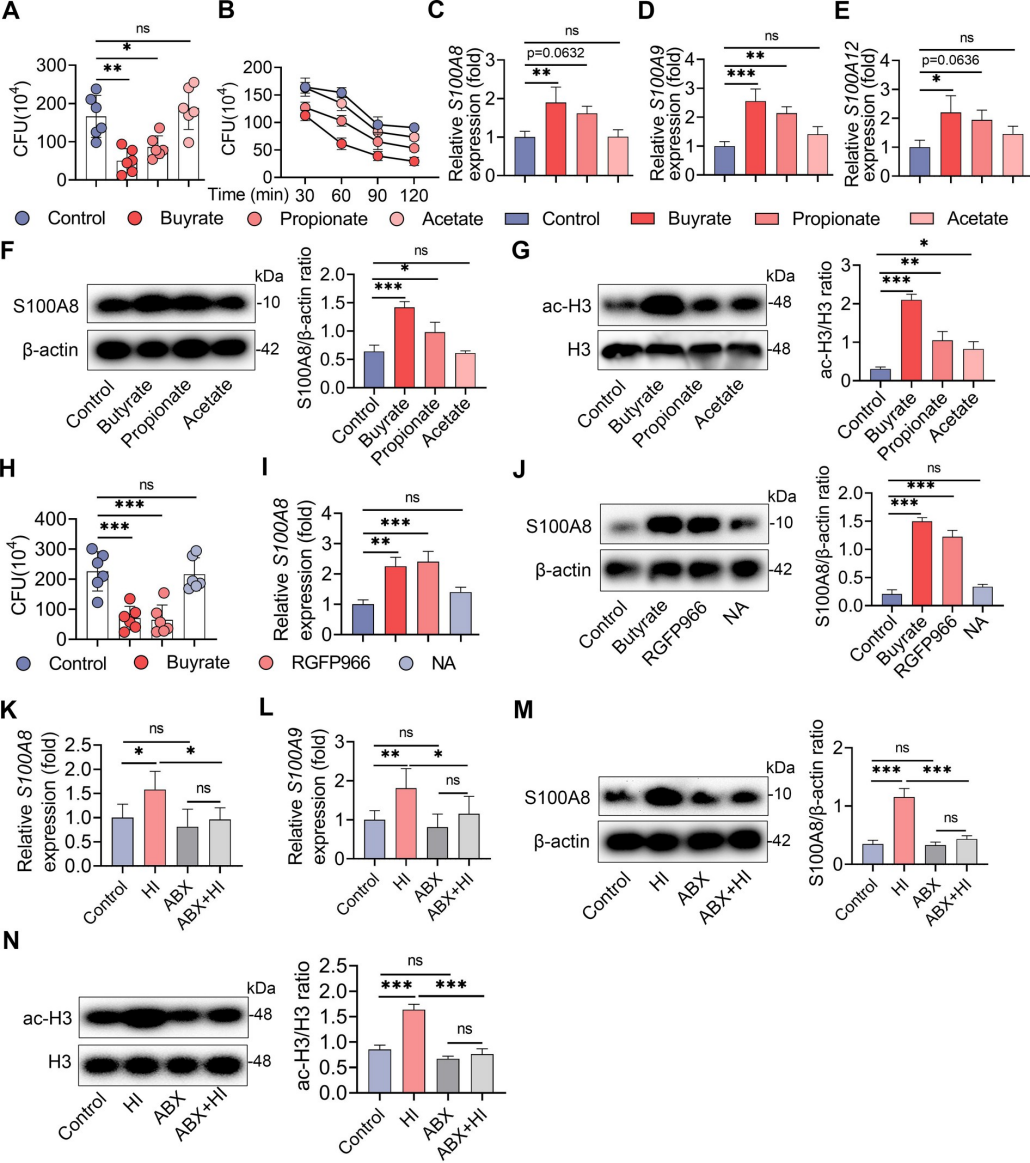
The HI diet activates macrophages antimicrobial defense through SCFA-mediated HDAC inhibition. Mice were treated with 30 mg/kg butyrate, propionate and acetate for a week, and then macrophages were isolated **(A-G**). **A**. Macrophages were infected with *S*. *au* (MOI of 10) for 1 h and intracellular bacterial loads were detected by gentamicin protection assay (n = 6). **B**. Intracellular bacterial loads in macrophages at different times after infection. **C-E**. Relative gene expression of *S100A8*, *S100A9* and *S100A12* in macrophages from different treatment groups by qPCR (n = 6). **F**. Relative expression of S100A8 antimicrobial protein in macrophages in different SCFA treatment groups. **G**. Expression of HDAC3 and acetylated H3 proteins in macrophages from the indicated groups. **H-I**. Mice were treated with butyrate, RGFP966 and NA for a week, and then macrophages were isolated (n = 6). **H**. Intracellular bacterial loads from indicated groups (n = 6). **I**. Relative gene expression of *S100A8* in macrophages from the indicated groups. **J**. Relative expression of S100A8 protein in macrophages in different treatment groups. **K-N**. Macrophages were isolated from the control diet or HI diet treatment groups with or without depletion of the gut microbiota. **K** and **L**. Relative gene expression of *S100A8* and *S100A9* in macrophages from different treatment groups by qPCR. **M**. Expression of S100A8 protein in macrophages in different groups. **N**. Expression of HDAC3 and acetylated H3 proteins in macrophages from the indicated groups. Data are presented as the means ± SD (**A-N**) and one-way ANOVA was performed for statistical analysis. *p < 0.05, **p < 0.01 and *** *p* < 0.001 indicate significant differences.

Since lower bacterial burdens were detected in the HI diet and butyrate and propionate treatment groups, we further focused on how the HI diet and SCFAs protect against *S*. *au* invasion. Our previous study found that butyrate, propionate and acetate did not limit the growth of *S*. *au in vitro* [5], which suggests that the reduced mammary *S*. *au* load in the butyrate treatment group was probably not attributed to the direct antibacterial effect of SCFAs. Other studies have reported that host cells, including macrophages, can limit pathogen invasion by activating antimicrobial immunity [23,24], which is also regulated by microbiota-derived SCFAs. Therefore, we next investigated the role of the antimicrobial defense of macrophages in HI diet-mediated protective effects against *S*. *au*-induced mastitis in mice. Macrophages isolated from mice in the butyrate and propionate treatment groups had reduced intracellular *S*. *au* compared with those of the control group (**Fig 7A**), while no significant difference was detected in the acetate treatment group (**Fig 7A**). Consistently, we found that faster clearance of intracellular bacteria was detected in macrophages isolated from butyrate- and propionate-treated mice (**Fig 7B**). However, acetate treatment had few effects on the clearance of intracellular bacteria compared with the control mice (**Fig 7B**). Previous studies have indicated that increased antimicrobial peptides are responsible for intracellular bacterial clearance in macrophages [23,25]. Indeed, SCFA treatment, particularly butyrate, significantly increased the gene expression of *S100A8*, *S100A9* and *S100A12* compared with the control treatment in macrophages (**Fig 7C–7E**). Likewise, increased S100A8 protein levels were detected in macrophages isolated from butyrate- and propionate-treated mice compared with control mice (**Fig 7F**). These results suggest that SCFA protects against *S*. *au* infection probably by activating the antimicrobial defense in the macrophages.

Given the greater protective effects and antimicrobial capacity of butyrate than propionate and acetate in this study, we next investigated whether histone deacetylase (HDAC) was responsible for the SCFA-regulated antimicrobial effect in macrophages as butyrate is a better HDAC inhibitor than propionate or acetate [23,26]. As expected, we found that macrophages isolated from different SCFA treatment groups had higher acetylated (ac)-H3 levels compared with the control macrophages (**Fig 7G**), where butyrate treatment led to a greater degree of ac-H3 expression (**Fig 7G**). Previous studies have also found that SCFA can limit *Salmonella typhimurium* infection by HDAC3-mediated antimicrobial defense [23]. Therefore, we examined whether the protective effect of butyrate is dependent on the inhibition of HDAC3 by treatment with the HDAC3 inhibitor RGFP966 and the HDAC inhibitor 1-naphthohydroxamic acid (NA) but without HDAC3 inhibition [27,28]. Similar to butyrate treatment, macrophages isolated from mice in the RGFP966 treatment group had a reduced intracellular bacterial load compared with those of the control group (**Fig 7H**). However, no significant change in the intracellular bacterial load was detected in the NA treatment group (**Fig 7H**). Likewise, increased antimicrobial defense was observed in microphages isolated from mice in the butyrate and RGFP966 groups, but not in the NA group, compared with the control group, as shown by higher levels of the *S100A8* gene and protein detected in the butyrate and RGFP966 groups (**Fig 7I and 7J**). These results suggest that SCFAs, particularly butyrate, activate antimicrobial defense in macrophages against *S*. *au* probably by inhibiting HDAC3.

Next we confirmed that macrophages isolated from the HI group had increased gene expression of *S100A8* and *S100A9* compared with those in the control group (**Fig 7K and 7L**), while these changes were reversed after the depletion of the commensal microbiota (**Fig 7K and 7L**). Consistently, higher S100A8 protein levels were detected in macrophages isolated from mice in the HI group than in those isolated from mice in the control group (**Fig 7M**), and this increase in S100A8 was eliminated after ABX treatment (**Fig 7M**). Moreover, we found that microphages isolated from mice in the HI group had increased ac-H3 protein levels compared with those in the control group (**Fig 7N**), while ABX treatment reduced this increase (**Fig 7N**). Collectively, these results suggest that an HI diet protects against *S*. *au*-induced mastitis by activating HDAC-mediated antimicrobial defense in macrophages through production of SCFAs.

## Discussion

Although the gut microbiota has been demonstrated to participate in the pathogenesis of mastitis [5–7], the potential effect of the modulation of the gut microbiota on pathogen-induced mastitis remains unknown. In the current study, we demonstrated that a fiber-enriched diet alleviates *S*. *au*-induced mastitis in a microbiota-dependent manner in mice by facilitating host antimicrobial immunity of macrophages. The underlying mechanism was involved in HDAC3 inhibition by production of SCFAs, particularly butyrate. Our findings confirm the important role of the gut microbiota in mastitis development and suggest that regulation of the gut microbial composition and function may serve as a potential strategy for infectious and inflammatory disease intervention.

Different dietary patterns are known as important regulators of the gut microbiota and subsequently influence the outcomes of diseases including infectious diseases. An et al., found that a Western-style diet (WD), characterized by high fat and low fiber contents, impeded the initial colonization and clearance of *Citrobacter rodentium* (*C*. *rodentium*) by regulating the gut microbiota [29]. However, supplementation with inulin regulated *C*. *rodentium* colonization in the context of WD [29]. Independent of the gut microbiota, WD also triggers systemic chronic and low-grade inflammation, which reduces the numbers of monocytes and neutrophils and promotes the aging of neutrophils, leading to exacerbated sepsis induced by LPS [30]. Similarly, short-term consumption of a high-fat diet increases host susceptibility to *Listeria monocytogenes* infection by altering the gut microbiota and increasing goblet cell number [18]. In addition, a high-salt diet compromises antibacterial neutrophil responses through hormonal perturbation and aggravates *Listeria monocytogenes* and *E*. *coli* infection [31]. Our previous study also showed that dietary tryptophan can limit *E*. *coli*-induced inflammation through activation of the aryl hydrocarbon receptor by the gut microbiota [6,32]. In the current study, we found that an inulin supplementation alleviated *S*. *au* infection in the mammary gland by changing the gut microbiome and producing SCFAs. Increased SCFA levels induced by microbiota-accessible carbohydrates or inulin can also suppress *Clostridium difficile* infection in mice [19]. A previous study showed that deprivation of dietary fiber was also found to promote susceptibility to *C*. *rodentium* infection in the gut by impairing the intestinal mucus and reducing SCFA levels [16,22]. In contrast, Mathis et al., reported that a low-fiber diet may contribute to protecting against *Listeria monocytogenes* and *Salmonella typhimurium* infection in the gut by increasing CD8^+^ T cells, Th1 cells and NK cells [33], suggesting that different pathogen types respond differently to the same dietary pattern.

Multiple manners are employed in gut microbiota-mediated antibacterial defense, among them is the production of SCFAs, which are often derived from a fiber-enriched diet via the gut microbiota. Osbelt et al., showed that variations in microbiota composition influenced *C*. *rodentium* infection by variable SCFA production [34]. Increased SCFAs can directly reduce virulence factors of pathogens, such as acylation of *Salmonella* pathogenicity island-1 (SPI-1), and thus attenuates infection [35]. In addition, SCFAs can increase the killing activity of macrophages by activating FFAR2 to protect against bacterial superinfection [36]. Propionate derived from the commensal microbiota can directly mediate colonization resistance to *Salmonella* and limit its growth by disrupting intracellular pH homeostasis [37]. Butyrate can also reduce bacterial infection by regulating macrophage homeostasis [38]. In this study, we showed that HI diet treatment increased SCFA levels and limited *S*. *au* infection in the mammary gland. Similar to previous studies [5,23], we showed that butyrate had better effects on *S*. *au*-induced mastitis and better antibacterial effects, which suggests that the underlying mechanism of SCFA on infection may involve different functions of host receptor activation. Indeed, apart from the direct activation of GPCR, SCFA can serve as a HDAC inhibitor with different affinities. Consistent with previous finding [23], we found that butyrate had a greater capacity to limit HDAC than propionate or acetate, accompanied by consistent protective effects in *S*. *au* infection. Previous studies have also reported that butyrate can induce a better antimicrobial program than acetate and propionate, which is associated with HDAC-mediated glycolysis and autophagy [23]. Likewise, increased HDAC expression was also identified in HI diet-treated mice, and this change was dependent on the gut microbiota and SCFA production. These results indicate that the antimicrobial program in macrophages induced by SCFAs, especially butyrate, is responsible for the protective effects of an HI diet on *S*. *au*-induced mastitis in mice. Notably, our results do not rule out the beneficial role of SCFA on host immunity, particularly inflammation inhibition [39], and other host receptor-regulated effects in the development of *S*. *au*-induced mastitis during HI diet supplementation. For example, SCFA, particularly butyrate, can limit the activation of NF-κB and NLRP3 pathways, which were involved in the pathogenesis of mastitis and responsible for the production of inflammatory cytokine [5,39,40]. Hence, multiple mechanisms may work synergistically to protect against pathogen-induced inflammation.

In conclusion, our results demonstrate that a fiber-enriched diet alleviates *S*. *au*-induced mastitis through producing SCFAs by the gut microbiota. The underlying mechanism involves the activation of the antimicrobial program in macrophages by SCFA-mediated HADC activation. Our findings suggest that regulation of the gut microbiota to facilitate SCFA production is a potential strategy to protect against pathogen-induced mastitis and other infectious diseases.

### Limitations of the study

Although our research provided evidence that a high-fiber diet alleviates *S*. *au*-induced mastitis in mice by SCFA-mediated antimicrobial program in macrophages, it is possible that other potential mechanism including regulation of inflammatory responses and host immune homeostasis may be involved in this progress. In addition, the role of a high-fiber diet in pathogen infections in distal organs needs to be further determined in humans.

## Materials and methods

### Ethical statement

The animal experiments were approved by the Institutional Animal Care and Use Committee (IACUC) of Jilin University (Changchun, China) (approval number: KT201903057). The full proposal was considered by the IACUC ethics committee, which approved the animal care and use permit license.

### Materials

Inulin was obtained from Yuanyebio Biotechnology Co., Ltd, (S11143, Shanghai, China). Ampicillin (Cat# A5354), neomycin (Cat# N6386), metronidazole (Cat# 16677), vancomycin (Cat# V2002) were bought from Sigma Aldrich (St. Louis, MO, USA). Specific antibodies including ZO-1 (1:1000; #AF5145; RRID: AB_2837631), Occludin (1:1000; #DF7504; RRID: AB_2841004), Claudin-3 (1:1000; #AF0129; RRID: AB_2833313) and β-actin (1:1000; #AF7018; RRID: AB_2839420) were purchased from Affinity Biosciences (OH, USA). S100A8 (#70802) and HDAC3 (#60538) antibody were bought from Cell Signaling Technology (Boston, USA). Acetylated-H3 antibody (Cat# ab47915; RRID: AB_873860) was purchased from Abcam (Cambridge, England). Tumor necrosis factor (TNF)-α (Cat# 430915) and interleukin (IL)-1β (Cat# 432615) enzyme-linked immunosorbent assay (ELISA) kits were obtained from Biolegend (San Diego, California, USA). Myeloperoxidase (MPO) (A044-1-1) assay kit was bought from Nanjing Jiancheng Bioengineering Institute (Nanjing, China,).

### Animals and experimental design

A total of 240 specific pathogen free (SPF)-grade BALB/c mice (180 female and 60 male, 21–24 g, 6–8 weeks old) used in the current study were obtained from Liaoning Changsheng Biotechnology Co., Ltd (Benxi, China). Mice were fed under SPF conditions with a 12-h light and dark cycle and supplemented with adequate water and food. After a week of acclimatization, three female mice and one male mouse were mated in an individually ventilated cage. Pregnancy was confirmed by vaginal plug and then male mice were removed.

The whole experimental design for this study included a high-fiber supplementation experiment, an antibiotic treatment experiment, a fecal microbiota transplantation (FMT) experiment, an SCFA supplementation experiment and an HDAC inhibitor supplementation experiment (Fig 1). For the high-fiber supplementation experiment, twenty-four mice that were pregnant for a week were randomly divided into four groups: (1) Control group: fed a control diet (AIN93G) for three weeks (n = 6); (2) HI group: fed a high-inulin diet (AIN93G + 20% inulin) for three weeks (n = 6) [41]; (3) *S*. *au* group: fed a control diet for three weeks followed by the *S*. *au*-induced mastitis model (n = 6); and (4) HI + *S*. *au* group: fed a high-inulin diet for three weeks followed by the *S*. *au*-induced mastitis model (n = 6). All these mice were fed different diets from one week after pregnancy, and mice were used for the *S*. *au*-induced mastitis model or PBS injection 7 days after parturition.

For the antibiotic treatment experiment, twenty-four mice that were pregnant for a week were randomly divided into four groups: (1) ABX group: mice were fed a control diet (AIN93G) and treated with an antibiotic cocktail (ABX) containing 1 g/L ampicillin, metronidazole and neomycin and 0.5 g/L vancomycin in drinking water for three weeks (n = 6); (2) ABX + HI group: mice were fed a high-inulin diet (AIN93G + 20% inulin) and treated with ABX for three weeks (n = 6) [41]; (3) ABX + *S*. *au* group: mice were fed a control diet and treated with ABX for three weeks followed by the *S*. *au*-induced mastitis model (n = 6); and (4) ABX + HI + *S*. *au* group: mice were fed a high-inulin diet (AIN93G + 20% inulin) and treated with ABX for three weeks followed by the *S*. *au*-induced mastitis model (n = 6). These mice were performed for the *S*. *au*-induced mastitis model or PBS injection 7 days after parturition.

For the FMT experiment, twenty-four pregnant mice were randomly divided into four groups: (1) CF group: mice underwent FMT for three weeks using mice in the control group as a donor (n = 6); (2) HIF group: mice underwent FMT for three weeks using mice in the HI group as a donor (n = 6); (3) CF + *S*. *au* group: mice underwent FMT for three weeks using mice in the control group as a donor followed by the *S*. *au*-induced mastitis model (n = 6); and (4) HIF + *S*. *au* group: mice underwent FMT for three weeks using mice in the HI group as a donor followed by the *S*. *au*-induced mastitis model (n = 6). FMT was performed according to previous studies [8,42]. In brief, fresh fecal samples were harvested from mice in the control and HI groups, and all fecal samples from the same group were mixed as a donor. The mixed fecal samples were thoroughly dissolved in sterile PBS (0.1 mg/mL) under anaerobic conditions. After centrifugation at 100 × g for 5 min, the supernatants containing microbiota were collected under sterile conditions. All pregnant mice used for the FMT experiment were first subjected to depletion of commensal microbiota by treatment with antibiotics (200 mg/kg ampicillin, metronidazole and neomycin and 100 mg/kg vancomycin) orally for five consecutive days [42]. After removing antibiotics for one day, each mouse that was pregnant for a week was given 300 μL of fecal microbial supernatant from the control or HI donors for three consecutive days and then once every two days for three weeks. At the end of FMT (7 days after parturition), the *S*. *au*-induced mastitis model was established in the CF + *S*. *au* and HIF + *S*. *au* groups. Mice in the CF and HIF groups were treated with an equal volume of PBS in the mammary glands.

For the SCFA supplementation experiment, thirty pregnant mice were randomly divided into five groups: (1) Control group: mice were treated with PBS orally for a week (n = 6); (2) *S*. *au* group: mice were treated with PBS orally for a week followed by the *S*. *au*-induced mastitis model (n = 6); (3) Butyrate *+ S*. *au* group: mice were treated with 30 mg/kg sodium butyrate orally per day for a week followed by the *S*. *au*-induced mastitis model (n = 6); (4) Propionate *+ S*. *au* group: mice were treated with 30 mg/kg sodium propionate orally per day for a week followed by the *S*. *au*-induced mastitis model (n = 6); and (5) Acetate *+ S*. *au* group: mice were treated with 30 mg/kg sodium acetate orally per day for a week followed by the *S*. *au*-induced mastitis model (n = 6). For the HDAC inhibitor supplementation experiment, twenty-four pregnant mice were randomly divided into four groups: (1) Control group: mice were treated with PBS orally for a week (n = 6); (2) Butyrate group: mice were treated with 30 mg/kg sodium butyrate orally per day for a week (n = 6); (3) RGFP966 group: mice were orally treated with RGFP966 (25 mg/kg) per day for a week (n = 6) [27]; and (4) NA group: mice were orally treated with 1-naphthohydroxamic acid (50 mg/kg) per day for a week (n = 6).

### Establishment of the mastitis model

A mouse mastitis model caused by *S*. *au* infection was established as previously described [5]. In brief, seven days after parturition mice were separated from the offspring for three hours and anesthetized with ethyl carbamate (100 mg/kg). The fourth nipple of the mammary glands of mice was sterilized with 75% alcohol and injected with 10^8^ colony-forming unit (CFU)/50 μL *S*. *au* using a 100-μL syringes with a 30-gauge blunt needle after anesthetized by ethyl carbamate (100 mg/kg). The control mice were anesthetized and treated with PBS. Twenty-four hours after *S*. *au* treatment, mammary tissues were collected and stored at -80°C until the detection of inflammatory parameters.

### Mammary bacterial burden assay

To investigate mammary *S*. *au* loads in different treatment groups, mammary tissues were harvested and weighed under sterile conditions. Mammary tissues were then prepared for 10% tissue homogenate using sterile PBS and 50 μL homogenate was plated on tryptic soy broth (TSB) agar plates. Twenty-four hours after incubation, the CFUs on the plates were counted and the bacterial load was calculated.

### Histological analysis

Mammary histological changes were determined using hematoxylin and eosin (H&E) staining as previously described [5,6]. Briefly, mammary tissues from different treatment groups were fixed with 4% paraformaldehyde for 48 h and prepared into 5-μm sections. After dewaxing and hydration using xylene and alcohol, the sections were stained with H&E. Histological changes were analyzed using optical microscopy (Olympus, Tokyo, Japan), and histological scores were assessed according to previous studies [5,6].

### Cytokine assay

To detect proinflammatory cytokine expression, mammary tissues from HI diet treatment groups, FMT groups and SCFA treatment groups were harvested and prepared as 10% homogenates using PBS. After centrifuging at 12000 × g for 10 min, the supernatants were collected and cytokines, including TNF-α and IL-1β, were detected using ELISA kits according to the manufacturer’s instructions (Biolengend, USA).

### MPO activity assay

As previously described [5], mammary tissues from HI diet treatment groups, FMT groups and SCFA treatment groups were collected and prepared as 10% homogenates and analyzed using an MPO assay kit according to the manufacturer’s instructions (Nanjing Jianchen, China).

### Bacterial culture

*S*. *au* ATCC 35556 was purchased from American Type Culture Collection (ATCC) and cultured in TSB medium (Hopebio, Qingdao, China) at 37°C for 12 h to reach logarithmic metaphase.

### Isolation of macrophages and gentamicin protection assay

For the gentamicin protection assay, macrophages were isolated from different treatment groups, including HI diet with or without ABX, SCFA, RGFP966 and NA treatment groups, as previously described [23,24]. Microphages from different groups were therefore treated with *S*. *au* (MOI of 10) for 1 h followed by gentamicin treatment (200 μg/mL) for 2 h. Cells were then lysed in 1% Triton buffer, and the lysate was plated on TSB agar plates. The results are presented as the absolute CFU count [23].

### Fecal total DNA extraction and 16S rRNA sequencing

Total microbial DNA from feces was extracted using a FastDNA Spin Kit for Soil (MP Biomedicals, U.S.) according to the manufacturer’s instructions and the hypervariable region V3-V4 of bacterial 16S rRNA was amplified by a specific primer (338F-806R). PCRs were performed in triplicate and the PCR product was extracted from a 2% agarose gel, purified using an AxyPrep DNA Gel Extraction Kit (Axygen Biosciences, Union City, CA, USA) according to the manufacturer’s instructions and quantified using a Quantus Fluorometer (Promega, USA). Purified amplicons were pooled in equimolar amounts and paired-end sequenced on an Illumina MiSeq PE300 platform/NovaSeq PE250 platform (Illumina, San Diego, USA) according to the standard protocols by Majorbio Bio-Pharm Technology Co. Ltd. (Shanghai, China). OTUs with a 97% similarity cutoff were clustered using a UPARSE version 7.1, and chimeric sequences were identified and removed. The taxonomy of each OTU representative sequence was analyzed by RDP Classifier version 2.2 against the 16S rRNA database using confidence threshold of 0.7. PCoA was performed to identify microbial structures and LEfSe was performed to identify bacterial taxa that were differentially enriched in different treatment groups. Shannon, Chao 1 and ace indices were performed for alpha diversity analysis. A Wilcoxon rank-sum test (FDR < 0.05) was performed to identify the differential bacterial taxa between the two groups.

### Fecal SCFA determination

Fecal SCFA concentrations were determined as previously described [5,43]. In brief, fecal samples were mixed with 50 μL 15% phosphoric acid, 100 μL isohexic acid (125 μg/mL) and 400 μL ether for 1 min, and centrifuged at 12000 × g at 4°C for 10 min. The supernatant was collected for testing using gas (Trace 1300, Thermo) and mass (ISQ 7000, Thermo) chromatography. The concentration of SCFAs was calculated according to the standard curve.

### RNA extraction and qPCR

Tissue samples were collected and total RNA was extracted using TRIzol (Invitrogen, CA, USA) as previously described [6]. cDNA was reverse transcribed using TransStart Tip Green qPCR SuperMix (TransGen Biotech, Beijing, China) and reacted with specific primers using FastStart Universal SYBR Green Master Mix (ROX; Roche, Switzerland, Basel) in a Step One Plus apparatus (Applied Biosystems, Foster City, CA, USA). Specific primers used in this study as follow: *S100A8* (sense) 5′-AAATCACCATGCCCTCTACAAG-3′ and (antisense) 5′-CCCACTTTTATCACCATCGCAA-3′; *S100A9* (sense) 5′- ATACTCTAGGAAGGAAGGACACC-3′ and (antisense) 5′- TCCATGATGTCATTTATGAGGGC-3′; *S100A12* (sense) 5′-CTTCCACCAATACTCAGTTCGG-3′ and (antisense) 5′-GCAATGGCTACCAGGGATATG-3′; and *GAPDH* (sense) 5′-AACTTTGGCATTGTGGAAGG-3′ and (antisense) 5′-ACACATTGGGGGTAGGAACA-3′. *GAPDH* served as an endogenous control and the 2^−ΔΔCt^ method was performed to calculate the relative expression of genes using the control as the calibrator.

### Western blotting

Western blotting was performed according to previously described methods [6]. In brief, the total proteins from the mammary gland samples were extracted by a tissue protein extract (Thermo Fisher Scientific, USA). Ten percent or 15% SDS-PAGE was performed followed by transfer to 0.45 μm PVDF membranes. After blocking with 5% skim milk for three hours at 37°C, specific antibodies at a final concentration of 1:1000, including ZO-1, Occludin, Claudin-3, S100A8, ac-H3 and β-actin, were incubated overnight at 4°C. The PVDF membranes were then incubated with goat anti-rabbit or rabbit anti-mouse IgG (1:20000) and analyzed using the ECL plus western blotting detection system (Tanon, China) after washing with TBST.

### Statistical analysis

GraphPad Prism 8.0 was used for the statistical analysis. Data are expressed as boxplots or the mean ± SD and representative data are one out of three independent experiments. Significant differences were evaluated using Mann-Whitney *U* test and one-way analysis of variance (ANOVA) followed by Tukey’s test. *p < 0.05 indicates significant difference. Other specific statistical analyses are described in the relevant methods section. The numerical data used in all figures are included in S1 Data.

## Supporting information

S1 Fig The effect of HI diet on alpha diversity of the gut microbiota in mice. Mice were fed a control diet or HI diet for three weeks and the gut microbiota were analyzed by 16S rRNA sequencing. **A** and **B.** Chao 1 and ace indices were shown. Data are expressed as boxplots and the Mann-Whitney *U* test was performed for statistical analysis (**A** and **B**).
(TIF)

S2 Fig FMT from the HI diet treatment group alters the gut microbiota in recipient mice. Pregnant mice were treated with ABX to deplete the commensal microbiota and then subjected to FMT from the control or HI treatment groups for two weeks (n = 6). **A** and **B.** Chao 1 and ace indices were shown. **C**. Gut microbial composition at the genus level from different treatment groups (n = 6). Data are expressed as boxplots and the Mann-Whitney *U* test was performed for statistical analysis (**A** and **B**). **p < 0.01 indicates significant difference.
(TIF)

S1 Data Excel spreadsheet containing, in separate sheets, the data points presented in Figs 1–7 and S1 and S2 Figs. (XLSX)
