# Supplementary figures and images for: A fiber-enriched diet alleviates Staphylococcus aureus-induced mastitis by activating the HDAC3-mediated antimicrobial program in macrophages via butyrate production in mice
Source: PLoS Pathog. 2023 Jan 19;19(1):e1011108. doi: 10.1371/journal.ppat.1011108 (PMC9888710; doi:10.1371/journal.ppat.1011108)

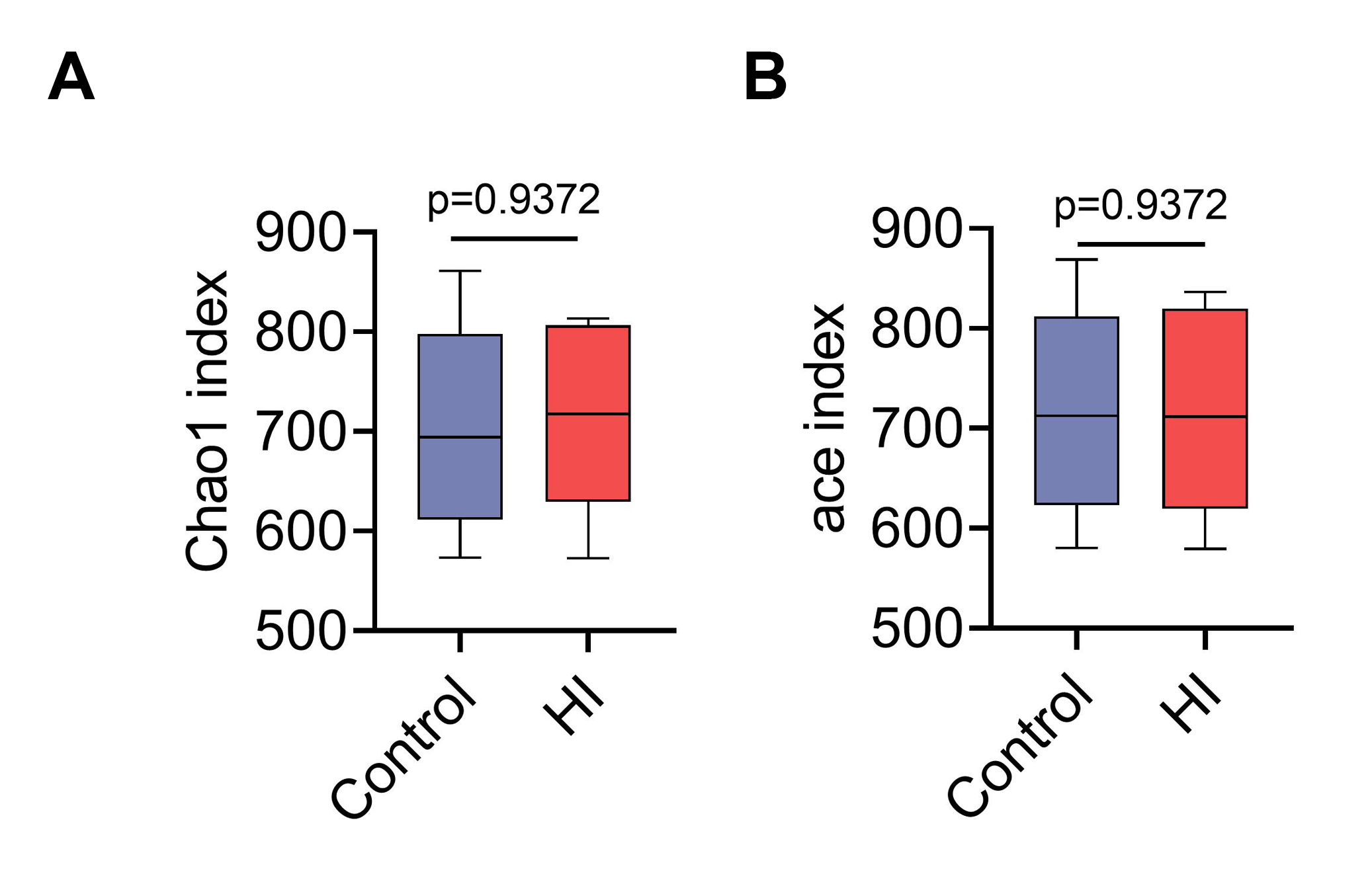

Supplement: S1 Fig — Mice were fed a control diet or HI diet for three weeks and the gut microbiota were analyzed by 16S rRNA sequencing. A and B. Chao 1 and ace indices were shown. Data are expressed as boxplots and the Mann-Whitney U test was performed for statistical analysis (A and B). (TIF) [file ppat.1011108.s001.tif]

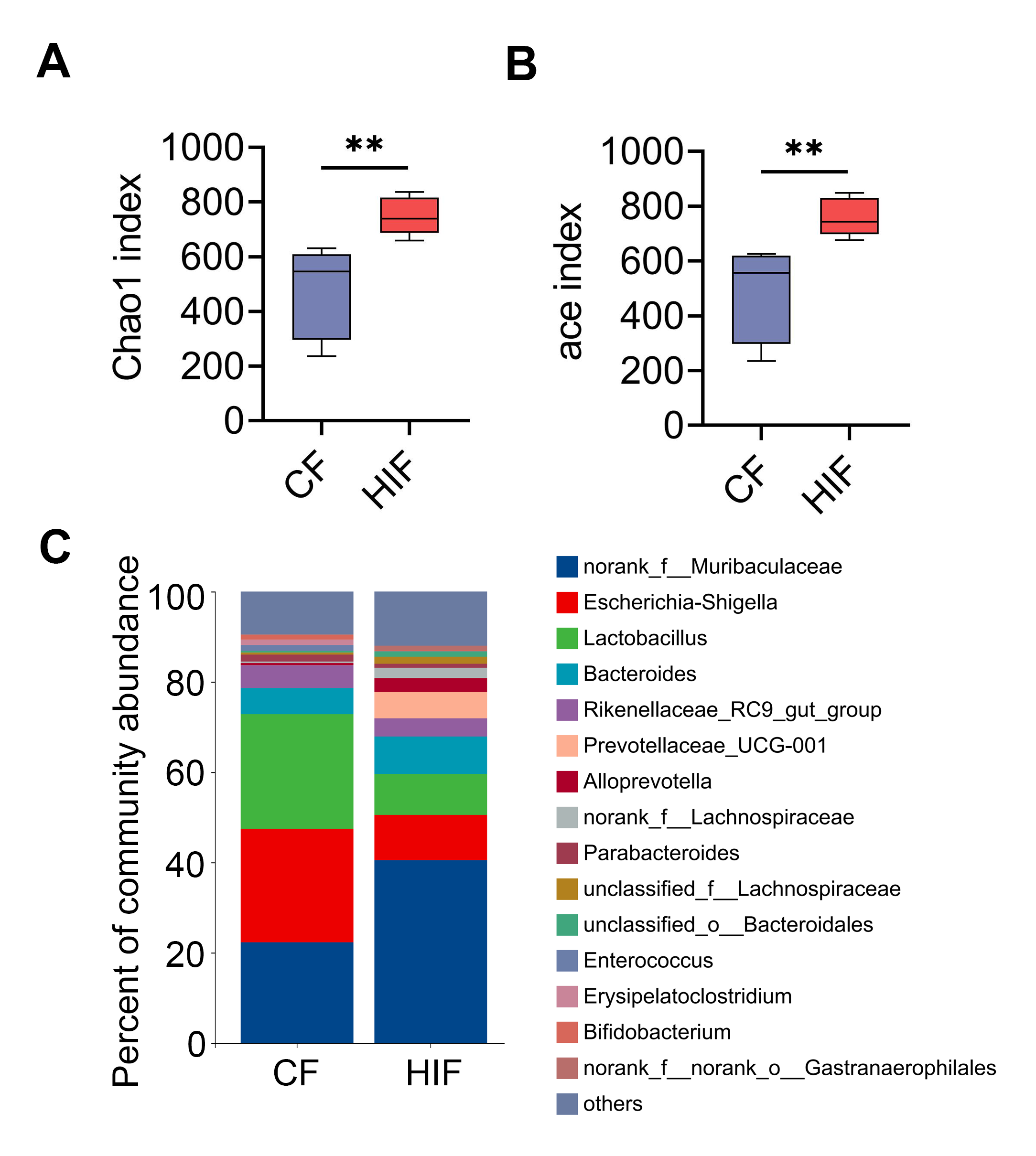

Supplement: S2 Fig — Pregnant mice were treated with ABX to deplete the commensal microbiota and then subjected to FMT from the control or HI treatment groups for two weeks (n = 6). A and B. Chao 1 and ace indices were shown. C. Gut microbial composition at the genus level from different treatment groups (n = 6). Data are expressed as boxplots and the Mann-Whitney U test was performed for statistical analysis (A and B). **p < 0.01 indicates significant difference. (TIF) [file ppat.1011108.s002.tif]
